# How plant neighborhood composition influences herbivory: Testing four mechanisms of associational resistance and susceptibility

**DOI:** 10.1371/journal.pone.0176499

**Published:** 2017-05-09

**Authors:** Tania N. Kim

**Affiliations:** Department of Biological Science, Florida State University, Tallahassee, Florida, United States of America; Helmholtz Zentrum Munchen Deutsches Forschungszentrum fur Umwelt und Gesundheit, GERMANY

## Abstract

Neighboring plants can decrease or increase each other’s likelihood of damage from herbivores through associational resistance or susceptibility, respectively. Associational effects (AE) can transpire through changes in herbivore or plant traits that affect herbivore movement, densities, and feeding behaviors to ultimately affect plant damage. While much work has focused on understanding the mechanisms that underlie associational effects, we know little about how these mechanisms are influenced by neighborhood composition, i.e., plant density or relative frequency which is necessary to make predictions about when AE should occur in nature. Using a series of field and greenhouse experiments, I examined how plant density and relative frequency affected plant damage to *Solanum carolinense* and four mechanisms that underlie AE; (i) accumulation of insect herbivores and arthropod predators, (ii) microclimate conditions, (iii) plant resistance, and (iv) specialist herbivore preference. I found a positive relationship between *S*. *carolinense* damage and the relative frequency of a non-focal neighbor (*Solidago altissima*) and all four AE mechanisms were influenced by one or multiple neighborhood components. Frequency-dependence in *S*. *carolinense* damage is most likely due to greater generalist herbivore load on *S*. *carolinense* (through spillover from *S*. *altissima*) with microclimate variables, herbivore preference, predation pressures, and plant resistance having relatively weaker effects. Associational effects may have long-term consequences for these two plant species during plant succession and understanding context-dependent herbivory has insect pest management implication for other plant species in agriculture and forestry.

## Introduction

Neighboring plants can decrease or increase the likelihood of damage to a focal plant compared to plants grown alone and these phenomena are known as associational resistance or susceptibility, respectively [1,2]. The topic of associational effects (AE) has a long history in applied fields such as agriculture and forestry [2] and previous empirical and theoretical studies suggest that AE can transpire through neighboring plant effects on both plant traits (such as plant nutritional quality and defenses) and herbivore traits (such as feeding behavior and movement) to affect herbivore densities and subsequent damage to plants [3,4]. While much effort has been put towards understanding the mechanisms that underlie AE (reviewed by [3]), challenges still exist in predicting when AE should occur in nature under different neighborhood conditions. This is because we have little understanding of how AE mechanisms relate to plant density and relative frequency which makes it impossible to predict the occurrence and magnitude of AE in nature. Furthermore, recent interest in understanding the long-term consequences of AE for plant competition and coexistence has emerged [3,5–8], therefore understanding how damage relates to plant density and relative frequency has community-wide implications.

**Fig 1:**
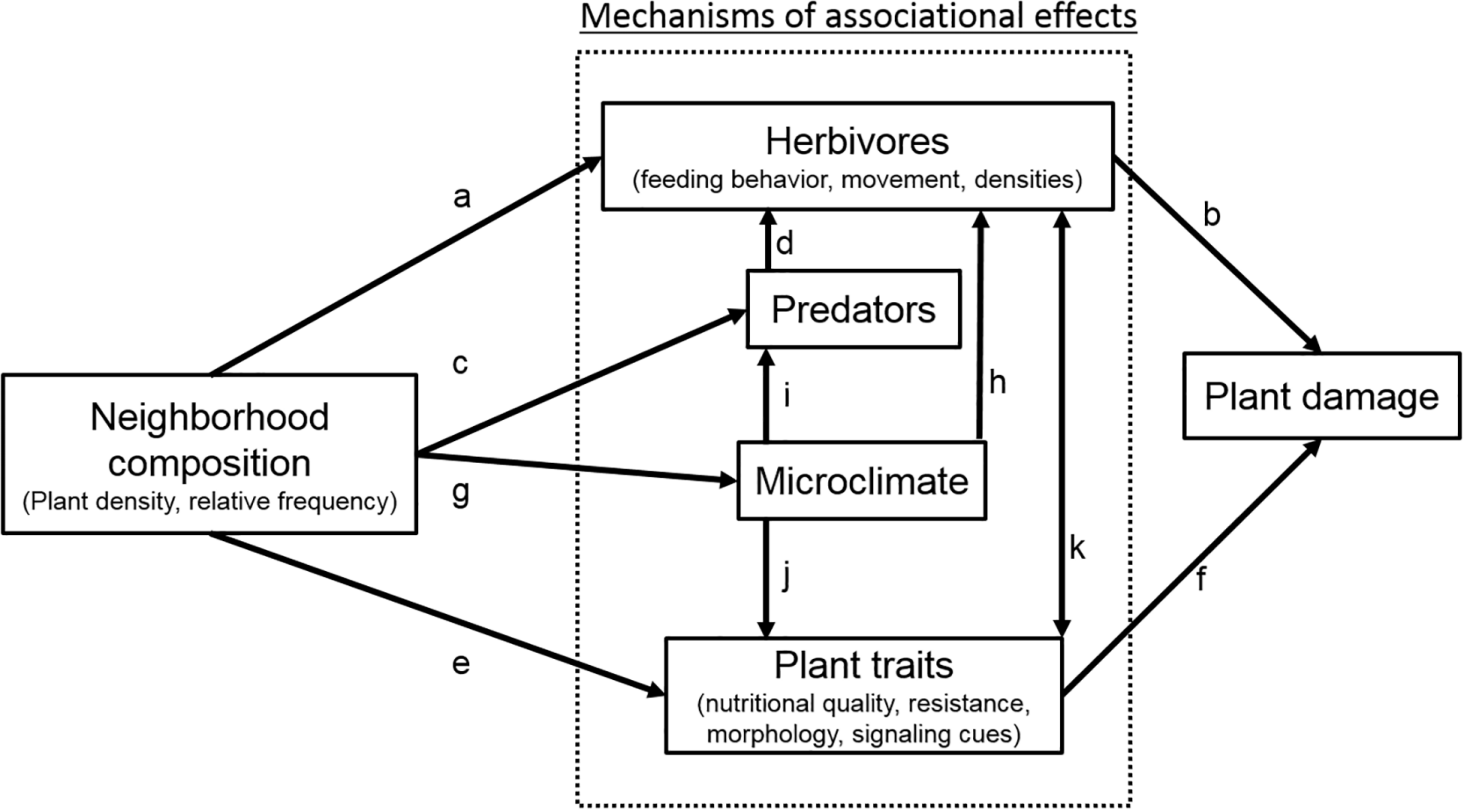
Mechanisms by which plant neighborhood composition affects plant damage. Mechanisms (in grey box) include plant neighborhood effects on herbivore traits and load (pathway a), predator load (pathways c and d), plant traits (nutritional quality, resistance, morphology, and signaling cues, pathway e), and/or microclimate conditions (pathways g, h, i, and j). Neighborhood mediated changes in plant and herbivore traits can lead to changes in plant damage patterns (pathways b and f). Feedbacks between plants and herbivores can occur (pathway k) to influence damage.

Numerous mechanisms have been proposed to explain how neighboring plants might influence damage on individual plants (reviewed by [3], Fig 1). AE can be mediated through changes in the characteristics of an individual herbivore (e.g., behavior, movement), herbivore populations (e.g., abundance or fecundity), or herbivore community structure (e.g., richness or composition). These mechanisms are likely to be influenced differently by the density (number of focal plants) and relative frequency of plants (proportion of non-focal plants) within the neighborhood (pathway “a” in Fig 1). For example, plant neighbors can influence a specialist herbivore’s ability to locate host plants by emitting volatile chemicals or visually masking host plants [9,10]. If a specialist herbivore is attracted to host plants that emit chemical or visual cues, then herbivores may be attracted to patches with a high density of these host plants leading to greater herbivore load and potentially greater plant damage (pathway “b”). On the other hand, if neighboring non-focal plants are also emitting chemicals that mask host plant cues, then host-plant selection by the specialist herbivore may be affected by the relative frequency of the non-focal plants rather than the density of the host plants per se. In this scenario, host-plants may experience associational resistance by non-focal plants that interfere host-plant signaling to the specialist herbivores [11]. For generalist herbivores that feed on a variety of different plant species, plant selection may depend on the total density of plants or the density of the preferred plant species. In this scenario, plants may incur damage through spillover effects from the preferred plant species resulting in associational susceptibility [11]. Neighboring plants can also affect herbivore and subsequent damage through their effects on predators (pathway “c”). For example, plant neighbors can attract predators by providing supplemental food or shelter resulting in consumptive or non-consumptive effects on herbivores, pathway “d” [12].

Other mechanisms of AE might be mediated through changes in plant traits such as plant nutritional quality, resistance, and morphology (pathway “e”, Fig 1) to affect herbivore movement and feeding behavior. For example, if resources are limited and the production of plant defenses is costly, then the presence of competitive neighbors could possibly constrain the production of plant defenses [13–18], making plants more vulnerable to herbivores and subsequent damage (pathway “f”). On the other hand, plant neighbors can facilitate one another by emitting volatile chemicals after being damaged that enable neighboring, undamaged plants to induce or prime the production of plant defenses [10,19–21]. Neighboring plants can also affect the abiotic environment such as temperature, light, and relative humidity (pathway “g”) thus influencing herbivore and predator feeding rates, development times, and their distributions, pathways “h” and “i” respectively [13,22,23] as well as plant growth patterns (pathway “j”). For example, shading by neighbors can alter plant morphology which could make focal plants more or less “apparent” [24–26] to insect herbivores.

While many studies have looked for the existence of AE [3,27], very limited knowledge exists of how mechanisms that underlie AE relate to the density and relative frequency of plants within a neighborhood (but see [4,28,29]) and how multiple AE mechanisms might contribute to damage simultaneously. While recent theorectical work suggests that plant density and relative frequency should affect plant and herbivore traits that will affect herbivore distribution and damage [4], we lack the necessary empirical data to know which components of the neighborhood (i.e., plant density and relative frequency) affect AE mechanisms (but see [28]). Traditional neighborhood studies have often used additive or substitutive experimental designs to manipulate neighbor and focal plant densities. These designs are problematic in that they confound effects of non-focal plant density with total plant density (additive designs, [30,31]), or confound host plant density with non-focal density (substitutive designs, [32,33]), making it difficult to assess the contribution of density or relative frequency effects on damage. These confounding issues can be overcome by adopting a response surface experimental design [34] that varies the both density and relative frequency of both species independently.

To my knowledge, no empirical study has addressed how the multiple mechanisms suspected to generate AE might change with the density and relative frequency of plant neighbors. Because different mechanisms may predominate at certain densities or interact with one another, examining multiple mechanisms simultaneously allows for a better understanding of how neighboring plants influence damage patterns and better predict the occurrence of AE in nature. Using a series of response surface experiments, I examined how neighborhood composition affects damage and four mechanisms that underlie AE. Specifically, I asked how the density and relative frequency of two perennial plants, *Solanum carolinense* and *Solidago altissima*, influenced damage and AE mechanisms: (i) insect herbivore and predator load, (ii) microclimate conditions including ambient temperature, light intensity, and soil water content, (iii) plant resistance, and (iv) herbivore preference. Here, density is the number of individuals within a standardized neighborhood area and relative frequency is the proportion of individuals of each species within each neighborhood. I also asked how these density- and frequency-dependent mechanisms relate to damage. Previous work in this system indicates that density and frequency can influence damage to *S*. *carolinense* differently [27] and herbivore effects can have long-term consequences for the plant community [35], however the mechanism(s) for how neighborhood composition influences damage remains unknown.

## Materials and methods

### Study system

I use *Solanum carolinense* (Carolina horsenettle, “*Solanum*”) as a focal species within neighborhoods of other *Solanum* individuals and *Solidago altissima* (Tall goldenrod, hereafter “*Solidago*”). I use *Solanum* as the focal plant species because it typically experiences a greater amount of, and variation in herbivore damage to leaves compared to *Solidago* which receives relatively little leaf damage at my field sites [35,36]. Both species are herbaceous perennials, native to the eastern US, and commonly found co-occurring in abandoned agricultural fields [37,38]. *Solanum* and *Solidago* support a diversity of insect herbivores including specialists (e.g. on *Solanum*: false potato beetle, *Leptinotarsa juncta* and tobacco hornworm *Manduca sexta*; on *Solidago*: goldenrod beetle, *Trirhabda virgata* and red goldenrod aphids *Uroleucon* sp.), and generalists (e.g. beet army worm, *Spodoptera exigua* and grasshoppers, *Melanoplus* and *Aptenopedes spp*.). Both plant species have physical and chemical traits suspected to deter herbivores [15,39] and herbivores are known to affect plant performance [40,41] and competitive interactions [35].

In 2011, I conducted three response surface experiments to examine the effects of neighborhood composition (i.e., plant density and relative frequency) on four mechanisms suspected to influence damage. For all three experiments, plants were clonally propagated from root cuttings (1.5g ± 0.2g each) taken from greenhouse grown plants collected from natural populations in north Florida between 2006 and 2011 (16 populations for *Solanum* and 10 populations for *Solidago*). In May 2011, each cutting was placed in a 530 mL nursery pot with a 3:1 mixture of Fafard 3 soil (Conrad Fafard Inc, Agawan, Massachusetts, USA) to coarse sand, and kept in a greenhouse at the Mission Road Research Facilities at Florida State University (Tallahassee, Florida, USA; 30.43° N, 84.28° W) for approximately five weeks (plant heights approximately 10–15 cm).

### Field experiment: Associational effects on damage, arthropods, and microclimate

**Fig 2:**
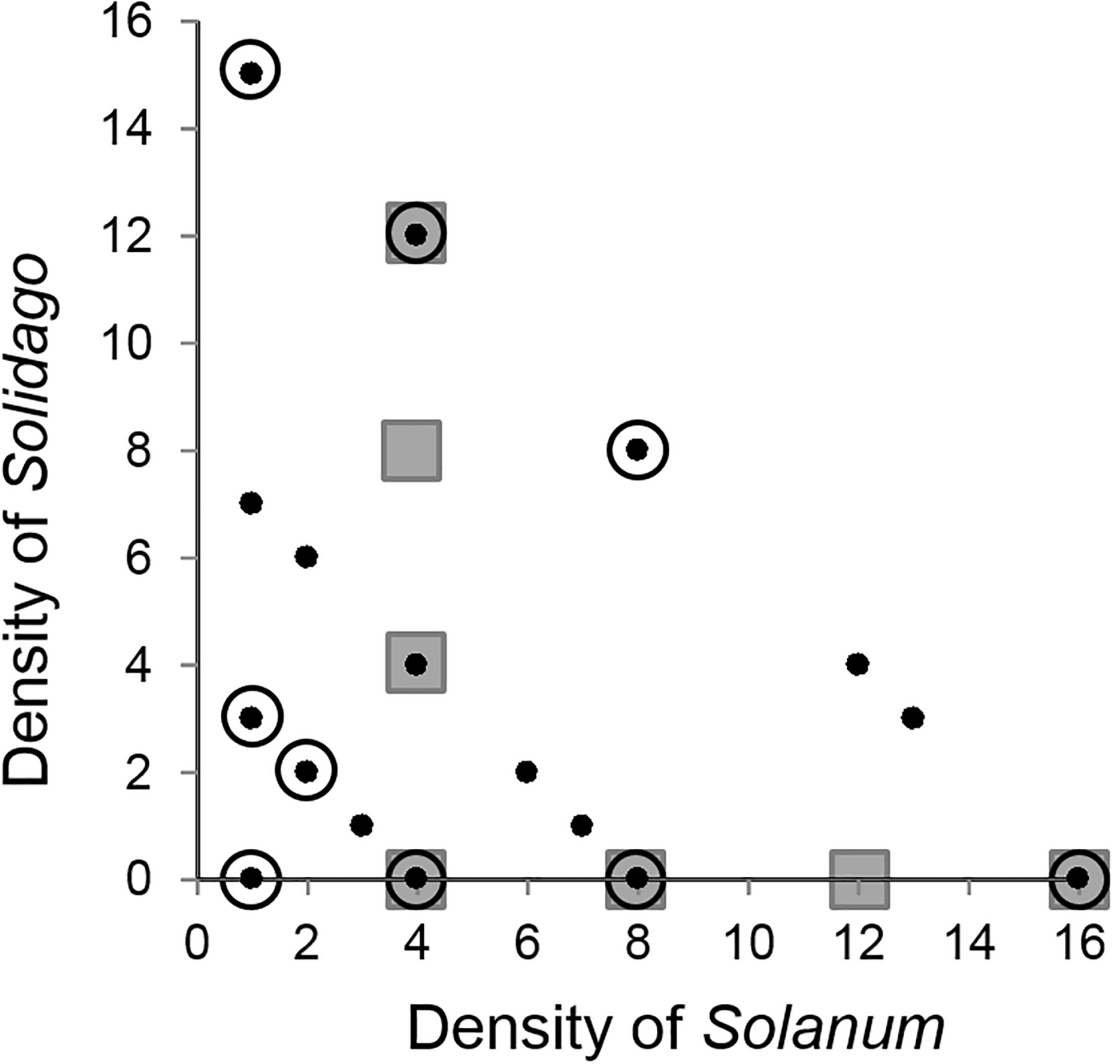
Response surface designs for three experiments. Densities of *Solanum carolinense* and *Solidago altissima* for field experiment (small black, filled circles) and herbivore preference experiment (large black, open circles). Values represent the number of plants per 91.4 cm diameter kiddie pools. Grey squares represent approximate densities of *Solanum* and *Solidago* for constitutive and induced resistance experiments in smaller pot sizes (45.7 cm diameter).

I examined how neighborhood composition influenced insect herbivore and predator loads, microclimate conditions, and *Solanum* damage by creating plant neighborhoods in plastic pools (91.4 cm diameter x 30.4 cm height, with added drainage holes). Neighborhoods within each pool contained 1, 4, 8, or 16 individuals per pool in varying combinations of *Solanum* and *Solidago* (small, black points in Fig 2). This translates to 1.57, 6.29, 12.59, and 25.19 plants per m^2^, respectively and these densities cover the natural range of densities in surrounding areas, 0.09 to 8.86 m^2^ (36). Each density combination (i.e., each point in Fig 2) was replicated three times, for a total of 483 plants in 51 pools. A random set of plant genotypes were used for this field experiment (13 genotypes for *Solanum* and 8 genotypes for *Solidago*)

I placed the pools in an abandoned agricultural field at the North Florida Research and Education Center (Quincy, Florida, USA; 30.58° N, 84.58° W) to allow natural colonization of insect herbivores and predators. Pools were arranged randomly into ~15 m diameter rings (eight to nine pools per ring, each pool separated by > two meters). A total of six rings were randomly placed in a 50m x 75m field and each ring was separated by 10–15 m. The vegetation between pools was largely composed of grasses (e.g., *Paspalum urvillei*, *Digitaria ciliaris*) with some herbaceous annuals and perennials (e.g., *Rubus trivalis*, *Sida spinosa)*. *Solanum* and *Solidago* were not observed in this particular field during the experiment but both species occur naturally in adjacent areas (at least 150 m away). *Solanum* and *Solidago* within the pools were visually surveyed for herbivores and predators between June and August (12 weeks). Each plant was surveyed two times per week (every three to four days) at different times during the day throughout the 12-week period to fully capture the herbivore and predator communities (N = 24 sampling dates). At the end of the growing season before leaf senescence (late September), leaf tissue damage (measured as percent leaf area removed) was visually assessed on all *Solanum* and *Solidago* leaves and averaged per individual.

To determine the effects of neighborhood composition on microclimate, three environmental variables were measured: ambient temperature, light intensity, and soil moisture. Data loggers (HOBO Pendant^®^) measuring light intensity (kilolumens/ m^2^) and temperature (degrees Celsius) were placed in the neighborhoods next to randomly selected *Solanum* plants (to measure light intensity) or under *Solanum* leaves (to measure temperature). Data loggers were placed out for three one-week periods in June, July, and August. Soil moisture was measured three times as well by collecting soil core samples (2.5 cm x 15 cm) in June, July, and August (two cores per pool per sample period). Soil samples were weighed wet (to the nearest gram), oven-dried at 60 degrees Celsius for one week, and re-weighed. The gravimetric water content, *Ɵ*_*g*_, was calculated as (wet mass-dry mass)/dry mass and soil water content was averaged per pool.

#### Statistical analyses for field experiment

The experimental unit was each “neighborhood” (i.e., each pool) therefore response variables were averaged across plants and growing season to yield one value per pool. I used multiple regression models (LMs) to examine the effects of total plant density, *Solanum* density, and frequency of *Solidago* (all fixed, continuous effects) on each of the following response variables: *Solanum* damage, herbivore and predator load per *Solanum* plant (the number of herbivores and predators per *Solanum* plant, respectively), ambient temperature, light intensity, and soil water content. *Solanum* leaf damage and arthropod load were log-transformed to meet the assumption of normality and homogeneity of variance of residuals in LM. In efforts to understand how neighborhood-mediated changes in arthropod load and microclimate variables relate to *Solanum* damage, I performed Pearson correlation tests between each of these response variables and *Solanum* damage. P-values were adjusted using Holm’s sequential Bonferroni correction [42] to control for family-wise error rates typically associated with multiple tests. All analyses were performed in R 3.1.0 [43].

### Greenhouse experiment: Associational effects on constitutive and induced plant resistance

To determine how neighborhood composition affects plant quality, I measured constitutive (CR) and induced (IR) resistance of *Solanum* to *Leptinotarsa juncta* (hereafter *“Leptinotarsa”*) across densities and frequencies of *Solanum* and *Solidago* in a greenhouse experiment in June 2011 (Mission Road Research Facility, Florida State University, Tallahassee, Florida, USA; 30.52°N, 84.4°W). *Leptinotarsa* was chosen because it is a specialist chrysomelid beetle known to feed on *Solanum* [41]. While *Leptinotarsa* is not numerically dominant in these fields compared to generalists such as grasshoppers, per-capita damage can be quite extensive (S. Halpern *unpublished data*). For this experiment, a second response surface set was created with a total of seven density combinations making up each set (gray filled squares, Fig 2). A subset of *Solanum* genotypes was used in this experiment (four genotypes in total) to account for genetic variation in defense and growth (D. McNutt *pers*. *comm*.). The four genotypes were a subset of the 13 genotypes used for the field experiment. To ensure equal genetic effects across neighborhood types, four complete sets were created, each response surface set was entirely composed of a single *Solanum* genotype. Each of the four response surface set (each with its unique *Solanum* genotype) was replicated twice (one set for CR analysis and the other set for IR analysis), resulting in a grand total of eight response surface sets (four genotypes x two sets per genotype) for the entire greenhouse experiment. *Solidago* clones were drawn from a random pool of 10 populations collected around north Florida. Plants were prepared using the same plant preparation protocol as above but rather than transferring plants into kiddie pools, plants were transferred into 45.7 cm diameter pots which were ¼ the volume of pools. The use of smaller volume pot sizes was necessary to accommodate experimental treatments in the greenhouse. The use of pools in the field would have required insecticide or netting to prevent colonization of herbivores which could have compromised the quality of leaves for bioassays. The numbers of plants per pot varied from one to four individuals, creating densities of 6.49 to 25.97 individuals per m^2^ which were comparable to field experiment described above. Plants were allowed to grow in competition for 6 weeks inside the pots before CR and IR were measured.

Resistance was measured as the relative growth rate (RGR) of *Leptinotarsa* larvae in laboratory bioassays. Because I did not want carry-over effects between the CR and IR experiments, different beetles were used for the bioassays. To measure IR, two randomly selected leaves on a randomly selected *Solanum* plant from each neighborhood in the IR response surface set were damaged at 30% using one to three *Leptinotarsa* larvae. Thirty percent leaf damage was a sufficient level to induce resistance in *Solanum* in previous studies (D. McNutt *pers*. *comm*.). Beetles were confined to leaves with mesh bags. Damage took less than 1 day after which beetles and bags were removed. After three days, the two most recently expanded undamaged leaves on the same plant were collected for bioassays with two 2^nd^-3^rd^ instar *Leptinotarsa* larvae per plant. Larvae were weighed (to the nearest milligram) after three hours of starvation to eliminate food in digestive tracts, and each beetle was offered one leaf in a 2.5 oz plastic cup (Dart Conex^®^) lined with damp filter paper. After 48 hours of feeding, *Solanum* leaves were removed. Beetles were starved for another three hours to eliminate food content in their guts [36] and weighed again. The RGR was calculated as ln (final mass / initial mass). To measure CR, two randomly selected undamaged leaves were removed from a randomly selected *Solanum* plant in each neighborhood in the CR (or control/undamaged) response surface set. Leaves were offered to two different 2^nd^-3^rd^ instar *Leptinotarsa* larvae and their relative growth rates were measured. To control for bag effects on leaf quality for IR plants, two leaves on these CR plants were bagged at the same time insects were bagged onto IR plants. CR was measured as simply 1- (RGR of herbivores fed CR plants). IR was measured as the 1- (RGR of beetles fed IR plants—mean RGR of beetles fed CR plants).

#### Statistical analyses for greenhouse experiment

The unit of analysis was each neighborhood type (i.e. each pot) so beetle responses per leaf and per plant were averaged. LMs were performed to examine the effects of total plant density, *Solanum* density, and the frequency of *Solidago* (all fixed, continuous effects) on resistance (CR and IR tests performed separately). Because plant genotype might influence damage levels, models also included plant genotype as a fixed effect (and two way interactions with total plant density, *Solanum* density and frequency of *Solidago*). Plant genotype was not included as a random effect because there were only four levels and mixed model estimation requires a minimum of five levels [44]. As with the field experiment above, I tested whether data met LM assumptions (e.g., residuals normally distributed, homogeneity of variance).

To determine how resistance might relate to damage in the field, I correlated damage with CR and IR using Pearson correlation tests (with a Bonferroni-Holm correction). I averaged CR and IR responses across plant genotype to determine the overall effect of plant neighborhood on CR and IR. Because these experiments were conducted in different settings (greenhouse versus field), I assumed that similar relative CR and IR responses to have occurred across the neighborhood treatments in the field.

### Behavior experiment: Herbivore foraging preference

Herbivores use a variety of sensory cues to detect and select host plants and plants (irrespective of their quality) can escape herbivory by reducing their “apparency” (sensu 24). In May 2012, I conducted a series of pair-wise preference tests using *Leptinotarsa* adults and a subset of nine neighborhoods from the field experiment (large, open, black circles Fig 2) to determine whether *Solanum* plants within different neighborhood types influenced *Leptinotarsa* preference. I focused on a specialist species, rather than a generalist species, because of clear preference for *Solanum* over *Solidago* and I focused on adults rather than larvae because host plant selection is typically performed by adults; larvae typically feed on plants where eggs were laid (T. Kim and D. McNutt *pers*. *obs*.). The same protocol for preparing plants was used except that individual plants remained in separate 530ml pots and neighborhoods were assembled just prior to data collection. This was done to prevent plants from competing within the same pots thus confounding effects of plant growth and plant quality, which would be a concern if competition between plants influenced plant size and quality. The sizes of neighborhoods were identical to those used for the field experiment and a random mixture of plant genotypes from different populations was used. Neighborhoods were paired and placed at opposite ends of an arena (2.5m x 1.25m x 0.75m in size, PVC framed and enclosed with plastic screening) with one *Leptinotarsa* adult placed in the middle of the arena between the neighborhoods. The locations of *Leptinotarsa* after 24 hours were recorded and preferences determined as the neighborhood where beetles were found or neighborhoods with signs of *Leptinotarsa* damage and eggs if beetles were not found on any plants. *Leptinotarsa* uses both visual and olfactory cues to detect plants from afar [25,45], therefore a period of 24 hours was used because it was long enough to allow beetles to acclimate to their new environments and select a patch from afar based on apparency (T. Kim *pers*. *obs*.), but short enough to prevent between-patch movement and assessing plant quality through contact cues.

I ranked neighborhoods based on *Leptinotarsa* preference. Because complete pairwise comparisons using all nine neighborhoods would require 36 different comparisons, a subset of 12 comparisons were made and replicated 10 times using different beetles for each replicate (120 preference trials in total). Four types of pair-wise comparisons were made to examine which component of the neighborhood (*Solanum* and *Solidago* density and frequency) influenced preference at (a) low total densities, (b) high total densities, (c) fixed *Solanum* densities, and (d) within *Solanum* monocultures (see S1 text for details).

#### Statistical analyses for behavior experiment

For each neighborhood type, the number of times that it was selected (or “wins”) was recorded and tallied. Neighborhoods differed in the number of times presented to beetles therefore I converted the total number of wins to proportion of wins. I used LMs to determine how total density, *Solanum* density, and *Solidago* frequency (all fixed, continuous effects) influenced the proportion of wins (arcsine-square root transformed). I tested whether data met LM assumptions (e.g., residuals normally distributed, homogeneity of variance). To determine how herbivore preference might affect damage in the field, I correlated damage measured from the field with preference measured from the greenhouse experiment using Pearson correlation tests (with a Bonferroni-Holm correction). Again, because these experiments were conducted in different settings (greenhouse versus field), I assumed that preference responses from the neighborhood treatments in greenhouse would also occur in the field.

## Results

### Associational effects on *Solanum* damage

**Fig 3:**
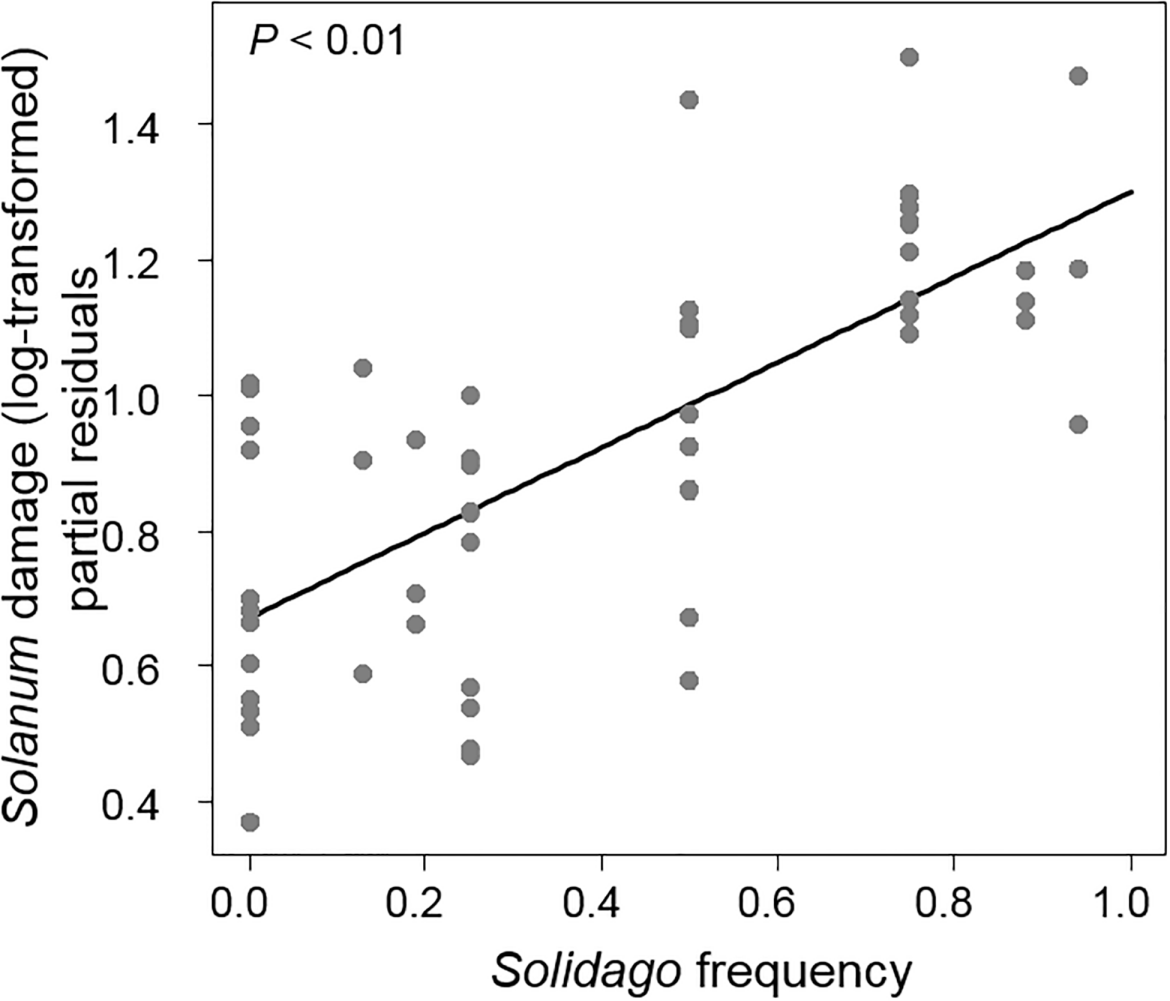
A positive relationship between the frequency of *Solidago* and *Solanum* leaf damage. Leaf damage estimated as the percent leaf area removed (log-transformed). Y values are partial residuals after accounting for the effects of the other neighborhood components.

The amount of damage to *Solanum* (mean = 13.7%; range = 1.2–43.1%) increased with the frequency of *Solidago* in the neighborhood, indicating associational susceptibility (*F*_1,47_ = 10.83, *P* < 0.01, Fig 3, Table A in S1 Table). There was no effect of total plant density (*F*_1,47_ = 1.15, *P* = 0.28) or *Solanum* density (*F*_1,47_ = 0.21, *P* = 0.64) on damage indicating no resource concentration or dilution effects. The amount of damage to *Solidago* was comparable to *Solanum* (mean = 11.7%, range = 5.3–24.1%) and *Solidago* damage followed similar patterns to *Solanum* where damage increased with the frequency of *Solidago* (*F*_1, 35_ = 6.74, *P* = 0.01) but not with total density (*F*_1,35_ = 0.07, *P* = 0.78) or *Solanum* density (*F*_1,35_ = 0.007, *P* = 0.93). All four mechanisms suspected to generate associational effects were affected by some aspect of neighborhood composition. A summary of neighbor effects is shown in Table 1.

**Table 1:** Neighborhood composition effects on mechanisms suspected to influence damage to *Solanum carolinense* (arthropod load, herbivore preference, microclimate, and resistance). The effects of total density, *Solanum* density and *Solidago* frequency are shown with standardized regression coefficients from linear models. The correlations between mechanisms and *Solanum* damage are shown with correlations coefficients, *R* (± 95% CI). Values in bold font are statistically significant at *P* = 0.05 with a Bonferroni-Holm correction.

Mechanisms	*Neighborhood composition components*			*R* (Lower, Upper 95%CI)
	Total density	*Solanum* density	*Solidago* frequency	
**(1) Arthropod load:**				
* -Herbivore load per Solanum*	-0.36	-0.15	0.05	0.14 (-0.13, 0.40)
* - Predator load per Solanum*	**-0.77**	0.24	0.35	0.04 (-0.23, 0.31)
**2) Herbivore preference**	-0.20	0.28	**-0.73**	**-0.91** (-0.98, -0.62)
**3) Microclimate:**				
* -Temperature*	**-0.68**	**0.61**	0.10	**-0.36** (-0.58, -0.09)
* -Light intensity*	**-0.58**	**0.77**	0.18	**-0.35** (-0.56, -0.07)
* -Soil moisture*	0.18	**-0.87**	**-1.16**	**-0.42** (-0.62, -0.15)
**4) Resistance:**				
* -Constitutive resistance*	-1.24	0.27	**0.74**	0.56 (-0.33, 0.92)
* -Induced resistance*	-0.75	0.19	**0.60**	0.51 (-0.38, 0.91)

### Associational effects on arthropod load

The herbivore community was largely composed of generalists such as grasshoppers *Melanoplus* and *Aptenopedes spp*, beetles such as weevils (*Sitonia* sp) and chrysomelids, and sucking insects such as the two-lined spittle bugs, *Prosapia bicincta*, meadow froghoppers *Philaenus spumarius*, making up 54% of the surveyed herbivores. *Solanum* specialists such as *Leptinotarsa*, *Epitrix fuscula* (eggplant flea beetle), and *Manduca sexta* (tobacco hornworm) made up 13.8% of the surveyed herbivores. Herbivores found on *Solidago* and *Solanum* substantially overlapped in community composition (59.6%), but over 60% of the herbivores surveyed were found on *Solidago* compared to *Solanum* (325 individuals versus 190 individuals, respectively). Herbivore load did not vary with any neighborhood component (Table B in S1 Table) but when herbivores were separated as generalists or *Solanum* specialists, specialist load (40% of *Solanum* herbivores) decreased with *Solidago* frequency (*F*_1,47_ 5.97, *P* = 0.02) while generalist load increased with *Solidago* frequency (*F*_1,47_ = 10.97, *P* < 0.01).

**Fig 4:**
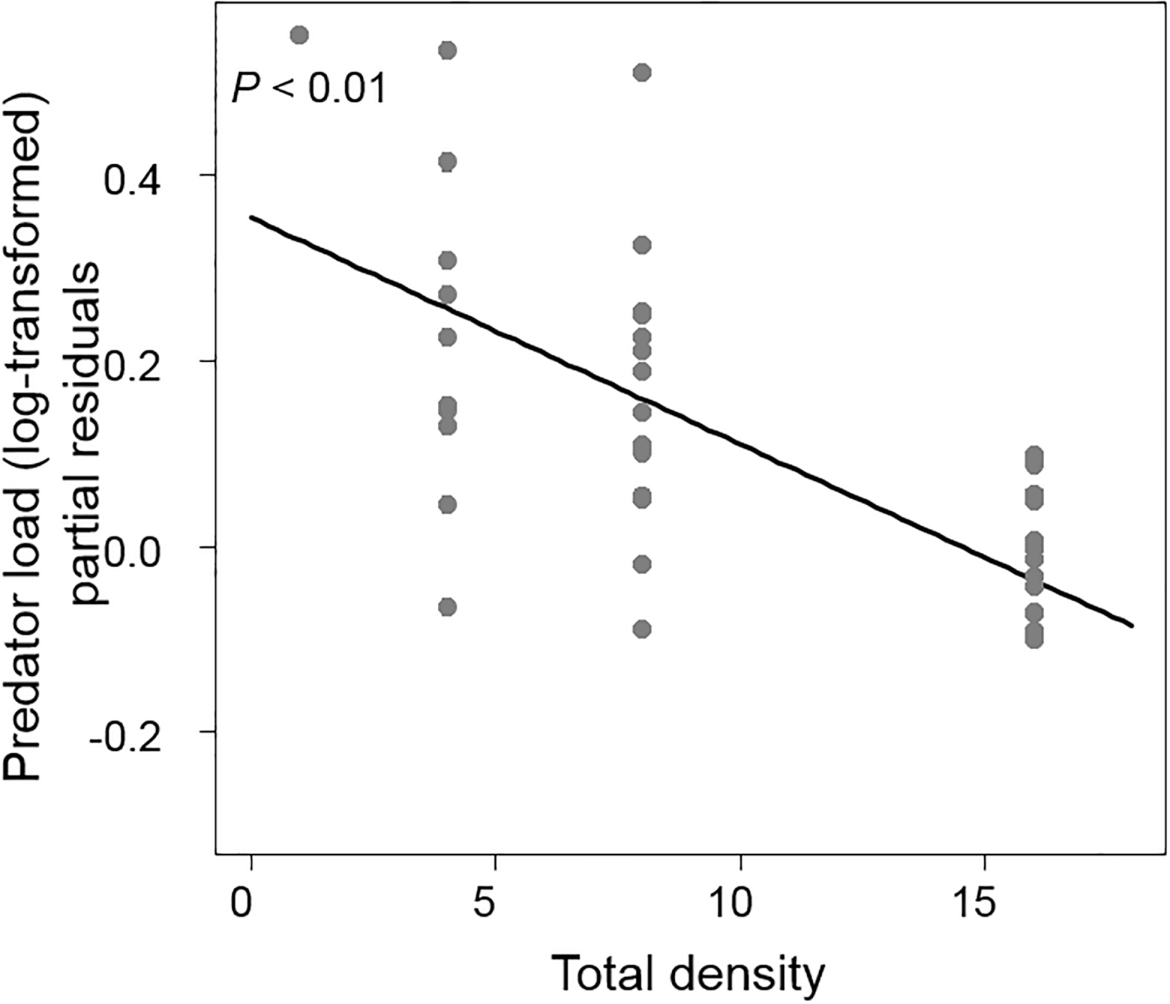
Total density effect on arthropod predator load on *Solanum*. Predator load was the number of predators surveyed on *Solanum* per *Solanum* plant (log-transformed). Y values are partial residuals after accounting for the effects of the other neighborhood components.

The predator community within the neighborhoods was largely composed of spiders (e.g. green lynx spiders *Peucetia viridians*, and yellow sac spiders *Cheiracanthium inclusum*) and predatory stinkbugs (e.g. *Euthyrhynchus floridanus* and *Stiretrus anchorago*) making up 80% of the surveyed individuals. Predator communities observed on *Solanum* and *Solidago* overlapped substantially (61.1% overlap) but most of the observed predators were found on *Solidago* compared to *Solanum* (340 individuals versus 87 individuals, respectively). Total plant density negatively affected predator load on *Solanum* (*F*_1,47_ = 8.32, *P* < 0.01, Fig 4, Table 1) with no effects of *Solanum* density and *Solidago* frequency (Table C in S1 Table). The ratio between predators and herbivore load on *Solanum* decreased with the total density (*F*_1,47_ = 6.48, *P* = 0.01, Table D in S1 Table) indicating that predation pressure decreased with increasing neighborhood size.

### Associational effects on microclimate

**Fig 5:**
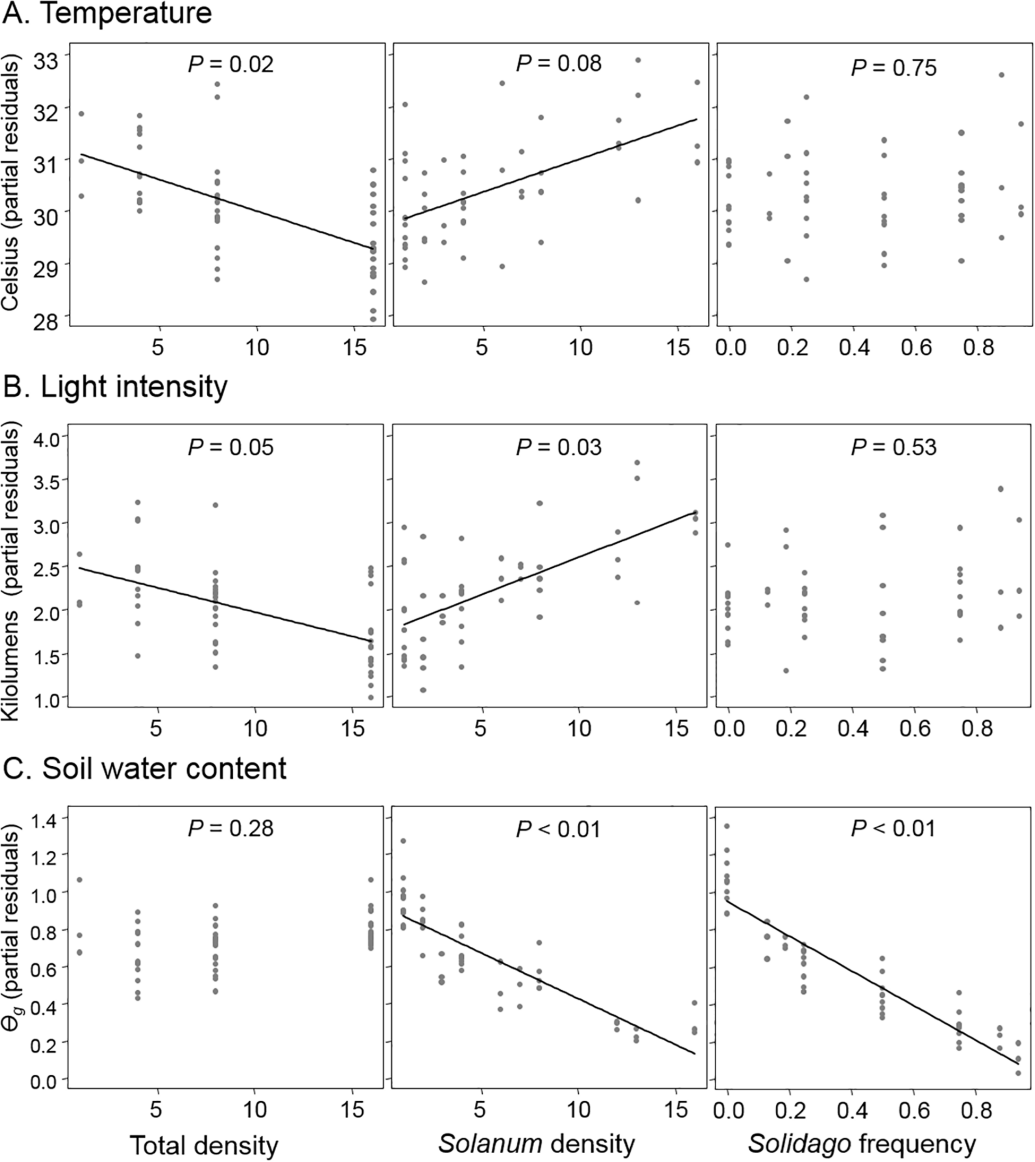
Neighborhood effects on microclimate variables: (A) temperature (degrees Celcius) (B) light intensity (kilolumens), and (C) gravimetric soil water content, Ɵ_g_ [(wet mass in grams-dry mass) /dry mass]. Y values are partial residuals after accounting for the effects of the other neighborhood components.

Neighborhood composition also affected microclimate variables (Table 1). The total density of plants negatively influenced mean temperature (*F*_1,47_ = 5.37, *P* = 0.02, Fig 5a, Table A in S2 Table) and light intensity (*F*_1,47_ = 3.78, *P* = 0.05, Fig 5b, Table B in S2 Table) where low density plots were 10% warmer and more light intense than high density plots. Light intensity also increased with *Solanum* density by 25% (*F*_1,47_ = 4.76, *P* = 0.03, Fig 5b, Table B in S2 Table). Finally, there was a negative relationship between soil water content and *Solidago* frequency (*F*_1,43_ = 53.55, *P* < 0.01, Fig 5c, Table C in S2 Table) and *Solanum* density (*F*_1,43_ = 18.68, *P* < 0.01). The mean soil water content was 60.65% in *Solanum* monocultures but 24.15% in neighborhoods dominated by *Solidago*.

#### Associational effects on plant resistance and herbivore preference using a specialist beetle

**Fig 6:**
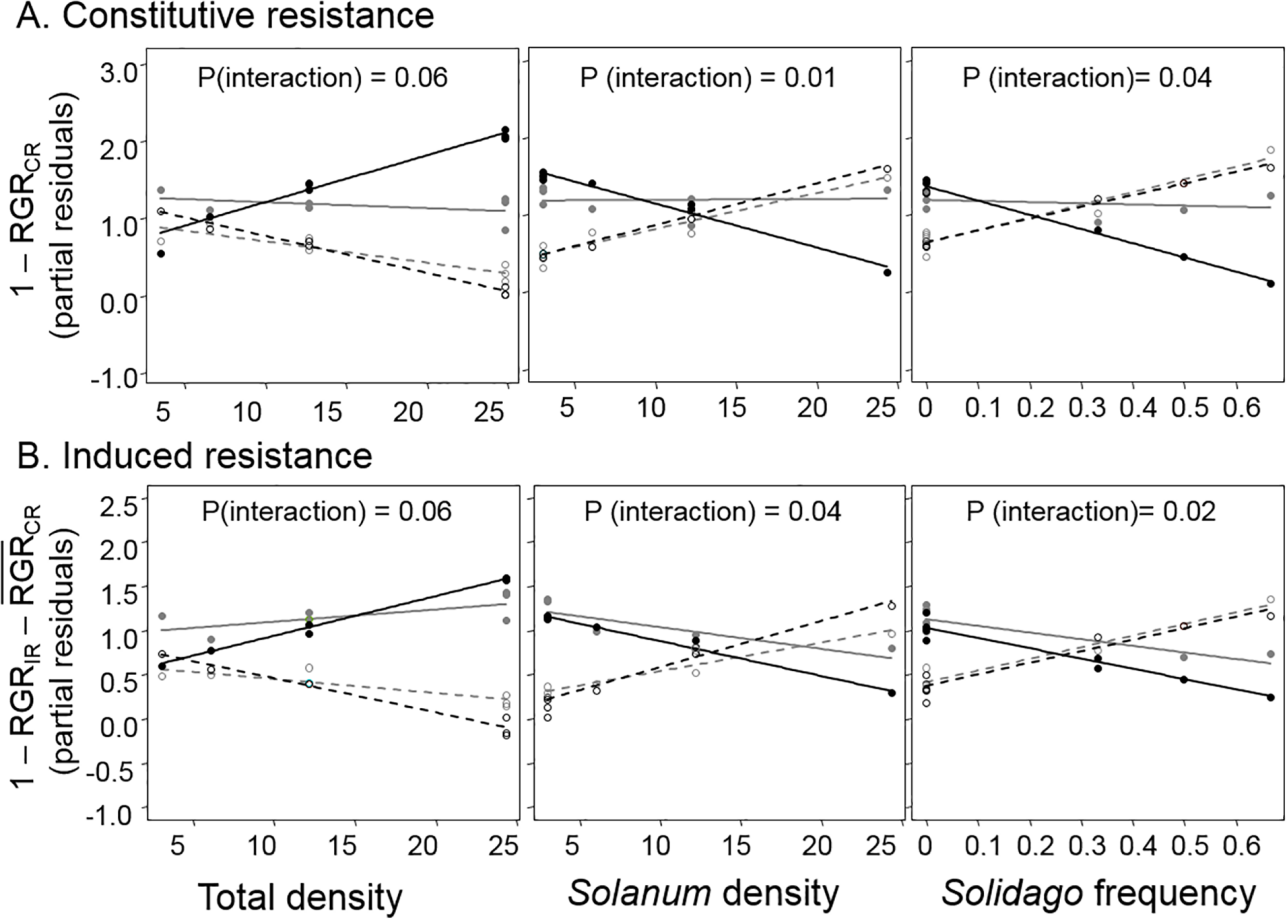
Effects of neighborhood composition on resistance: (A) constitutive resistance (CR) and (B) induced resistance (IR). Resistance was measured using the relative growth rates (RGR) of *Leptinotarsa juncta* beetle larvae (CR = 1 − RGR_CR_ and ${\text{IR}=1\text{ RGR}}_{\text{IR}}-\;{\bar{\text{RGR}}}_{\text{CR}}$). Solid grey and black lines/circles are for genotypes #111 and 5, respectively. Dashed grey and black lines and hallow circles are for genotypes #101 and 202, respectively. P-values are for interactions between the four plant genotypes and neighborhood components. Y values are partial residuals after accounting for the effects of the other neighborhood components.

Neighborhood composition influenced both induced and constitutive resistance of *Solanum* to *Leptinotarsa* larvae but relationships varied with plant genotype (Fig 6, Table 1, S3 Table). Specifically, *Solidago* frequency interacted with plant genotype to influence CR (*F*_3,12_ = 4.49, *P* = 0.04) and IR (*F*_3,12_ = 4.38, *P* = 0.02) and *Solanum* density interacted with genotype to influence CR (*F*_3,12_ = 3.22, *P* = 0.01) and IR (*F*_3,12_ = 3.56, *P* = 0.04). Although there were no general relationships between neighborhood composition and IR/CR among genotypes, three of the four genotypes (#5, 104, 202) consistently responded strongly to neighborhood composition (although in opposite directions), while one genotype did not respond to neighborhood composition (#111).

**Fig 7:**
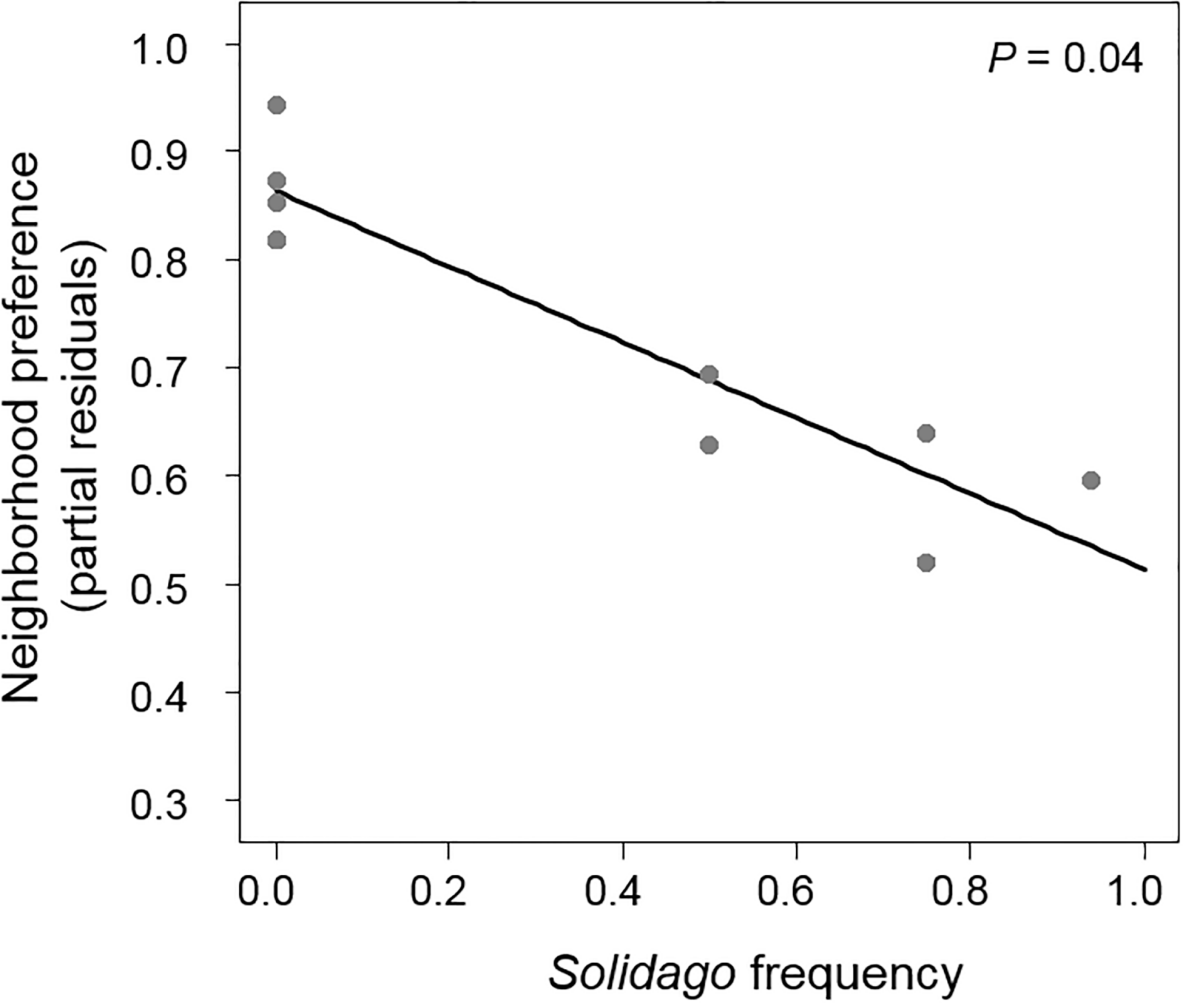
*Leptinotarsa juncta* beetles preferred neighborhoods with a lower frequency of *Solidago*. Preference determined as the proportion of times *L*. *juncta* selected a particular neighborhood type (arc-sine square-root transformed). Y values are partial residuals after accounting for the effects of the other neighborhood components

*Leptinotarsa* preference also varied with neighborhood composition (Table 1). Specifically, *Leptinotarsa* presence increased in neighborhoods with lower *Solidago* frequency (*F*_1,5_ = 6.96, *P* = 0.04, Fig 7) but did not vary with *Solanum* density (*F*_1,5_ = 1.45, *P* = 0.28) or total density (*F*_1,5_ = 0.99, *P* = 0.36). This suggests that *Leptinotarsa* did not prefer neighborhoods with a greater density of host plants *Solanum* per se but instead avoided neighborhoods with a dominated by *Solidago*.

### Relationships between AE mechanisms and damage

To determine how *Solanum* damage might be influenced by density- and frequency dependent mechanisms, I performed separate Pearson correlation tests with each of the measured response variables mentioned above (Table 1). I did not find any correlation between *Solanum* damage and total herbivore load per plant (*t* = 1.2, *df* = 49, *P* = 0.30) but when herbivores were separated by diet specialization, I observed a positive correlation between *Solanum* damage and generalist herbivore load (*t*_1,49_ = 3.09, *P* < 0.01, *r* = 0.4) and no correlation between damage and specialist herbivore load (*t*_1,49_ = -1.50, *P* = 0.13). There were strong negative correlations with damage and *L*. *juncta* preference (*t* = - 5.85, *df* = 7, *P* < 0.01, *r* = - 0.91) and microclimate variables (temperature: *t* = - 2.69, *df* = 48, *P* < 0.01, *r* = - 0.36; light intensity: *t* = -2.53, *df* = 48, *P* = 0.01, *r* = - 0.35; and soil moisture: *t* = - 3.19, *df* = 48, *P* < 0.01, *r* = - 0.42). I did not find any correlations between damage and predator load (*t* = 0.29, *df* = 49, *P* = 0.77) or resistance (CR: *t* = 1.52, *df* = 5, *P* = 0.18; IR: *t* = 1.34, *df* = 5, *P* = 0.23).

## Discussion

Neighborhood composition has long been recognized as an important predictor for plant performance [46] and decades of research, particularly in agriculture, demonstrate that different neighborhood types can influence attack rates by herbivores [3]. However, little progress has been made in identifying which components of the neighborhood (i.e. density and relative frequency of plants) influence damage and associated mechanisms (but see [27–29]). Determining the relationships between neighborhood components and AE mechanisms will allow us to predict the conditions under AE will occur and provide a better understanding of how and when herbivores might structure plant communities. In this study, damage on *Solanum* increased with *Solidago* frequency suggesting associational susceptibility and there were no density effects (either total or *Solanum* density). I found that all four mechanisms suspected to influence damage were affected by some component of the neighborhood (Table 1); however, plant frequency had an overall greater effect by influencing all four mechanisms. These frequency-dependent mechanisms may act alone or interact with one another to ultimately influence frequency-dependence in damage.

### Frequency-dependent mechanisms of damage

While all mechanisms were influenced by neighborhood composition, frequency-dependence in damage to *Solanum* was likely due to greater generalist herbivore load. *Solidago* is a tall, erect plant (mean height = 122 cm in the field) compared to *Solanum* and the other grasses and forbs in the area, and neighborhoods with greater *Solidago* frequency may have attracted generalist herbivores from the surrounding vegetation by providing an alternative food choice, refuge, or shelter [6,47]. Increased herbivore aggregation to these neighborhoods may have led to spillover of generalist herbivores to *Solanum* resulting in greater damage. Indeed, damage to the non-focal plant, *Solidago* was also positively influenced by *Solidago* frequency and the overall amount of damage to both plants species were comparable in magnitude (13.7% in *Solanum* and 11.7% in *Solidago*). Because there was great overlap in the herbivore communities on *Solanum* and *Solidago*, these results suggest that the same, generalist species were likely feeding on both plant species and contributing to frequency-dependence in damage.

Microclimate conditions within neighborhoods with greater *Solidago* frequency may have also contributed to increased damage and herbivore load by providing suitable abiotic conditions to complete insect lifecycles, and increase insect growth and reproduction rates [16]. For example, soil moisture can influence damage and herbivore load directly through effects on development time and feeding rates [48,49] or indirectly through changes in plant quality [50]. In this study, soil moisture was negatively correlated with damage suggesting that herbivores preferred neighborhoods with drier soils. Many insects found in this study pupate in soil thus neighborhoods with lower soil moisture may have provided ideal conditions for feeding or increasing reproduction (e.g., oviposition sites on nearby plants [16], faster pupation rates [23]). While the current study cannot determine the exact mechanism by which microclimate affects damage, we know that abiotic conditions can greatly influence plant and arthropod growth. Because virtually nothing is known about the direct and indirect contributions of microclimate to AE, future studies that manipulate microclimate variables and follow herbivore and predator growth, reproduction, and feeding strategies are needed [3].

In a behavior experiment, there was a strong negative relationship between *Solidago* frequency and the presence of *Leptinotarsa* (a specialist herbivore to *Solanum*). *Solanum* in these particular neighborhoods may have been less apparent than in neighborhoods with low *Solidago* frequency as *Solidago* could mask *Solanum* visually [25,51] or through scent [45]. Of the few *Solanum* specialists observed in the field, there was a negative relationship between the frequency of *Solidago* and specialist herbivore load (suggesting associational resistance) thus matching results from the behavior experiment. However, there was no correlation between *Solanum* damage and specialist herbivore load in the field and *Solanum* in neighborhoods with greater *Solidago* frequency received relatively more damage than in low *Solidago* frequency neighborhoods. Instead, damage was positively correlated with generalist load; generalist load increased with *Solidago* frequency. These results, in addition to the fact that generalist herbivores were by far numerically dominant compared to specialists, suggest that *Solanum* damage was largely due to generalist herbivores likely due to spillover from *Solidago* (indicating associational susceptibility). Conducting behavior experiments with generalists or tracking movement and damage patterns in the field will elucidate whether spillover is occurring. Previous work on associational effects has largely focused on mechanisms associated with specialist herbivores. Future studies should give greater consideration to the contribution of generalists (or shared herbivores) to damage patterns particularly if generalist and specialist herbivores are responding to neighborhood composition in different ways within the same system [28, 29].

Although associational effects can manifest through changes in plant resistance, there were no generalizable patterns with resistance and neighborhood composition, as the four plant genotypes used in this study responded differently to plant density and frequency. Furthermore, there was no general relationship between *Solanum* damage and the mean resistance levels of the all four plant genotypes combined. Previous work in this system has demonstrated that susceptibility to damage in the field varies dramatically with *Solanum* genotype (Kim, unpublished data) likely due to different levels of investment towards plant resistance and/or tolerance [52]. Therefore, the relationships between resistance and damage for each genotype could influence how different genotypes respond to plant competition (i.e., neighborhood composition). Future studies using larger samples of genotypes, increasing the strength of competition between plants (e.g. higher plant densities, run experiment longer) and relating damage patterns from the field could elucidate whether general patterns among different plant genotypes can be observed and whether resistance is an important mechanism for associational effects.

### Scaling up context-dependent herbivory

Distinguishing context-dependent herbivory as frequency- or density-dependent has important implications for both applied and basic ecology. From a basic ecology perspective, understanding the long-term consequences of damage for plant communities requires knowing how damage varies with plant density and frequency. *Solidago* and *Solanum* are two early successional old-field plants and without disturbance, old field plant communities become dominated by dense stands of *Solidago*. Results from this study indicate that increasing the frequency of *Solidago* can increase damage to *Solanum* (likely due to spillover) which could have long-term consequences for the persistence of *Solanum* in old-field communities. In a previous study in the same study system [27], we also observed frequency-dependent herbivory which affected competitive interactions between *Solanum* and *Solidago* and the likelihood of coexistence [35]. In particular, when herbivory was high, competitive exclusion was observed (*Solidago* excluded *Solanum*) but when herbivory was low, coexistence between *Solanum* and *Solidago* was observed. Because plant neighborhood composition can influence the likelihood of damage which can, in turn, feedback to influence plant neighborhood composition, understanding how AE changes with the neighborhood composition is critical for evaluating whether dynamic feedbacks are occurring between herbivores and plants and their contribution towards maintaining plant diversity patterns in old-fields [8] and other plant communities [53,54].

From an applied perspective, insect pest management in agriculture or forestry requires correct planting strategies to minimize insect load, damage, and insect spread. If damage was dependent on the density of the focal plant alone, then planting non-focal plants to diversity plantings would be an ineffective strategy to minimize damage by herbivores. However, if damage is indeed frequency-dependent, then considering the frequency of non-focal plants would be an essential component of pest management. Understanding AE mechanisms can also inform management as well. For example, if plant resistance was constrained by resource competition, then management strategies aimed at alleviating competition could be employed such as the use of fertilizer or increased spacing between plants. Alternatively, if mechanisms are mediated through herbivores or predators, then the use of trap crops or refuges could be used to minimize herbivore distribution onto focal plants.

This experiment was done at a small spatial scale; different mechanisms could be operating at larger spatial scales to influence damage. Host plant detection, selection, and subsequent damage by herbivores is a result of various, often hierarchical, mechanisms [4,55], therefore, it is possible that many of the mechanisms examined in this study predominate at different spatial scales. For example, at large spatial scales, total density of plants may be important as herbivore and predator immigration into patches might rely on patch size for easier detection from afar. However, once in the patch, host-plant apparency or plant quality could influence host-plant selection and damage which might be dependent on the host-plant density or resource competition. Because host-plant finding is a multi-scale process, understanding how herbivores select host-plants both from afar and once in the patch is necessary if we want to predict and compare the prevalence of frequency- and density-dependent mechanisms of damage. Thus special consideration should be given to selecting the appropriate spatial scale for a given plant-herbivore system [7].

## Supporting information

S1 Table ANOVA tables for neighborhood composition effects on *Solanum* damage, insect load, and predation pressure. (PDF)

S2 Table ANOVA tables for neighborhood composition effects on microclimate conditions. (PDF)

S3 Table ANOVA tables for neighborhood composition effects on plant resistance. (PDF)

S1 Text Neighborhoods used for pair-wise preference experiments. (PDF)

## References

[1] Tahvanainen JO, Root RB. The influence of vegetational diversity on the population ecology of a specialized herbivore, Phyllotreta cruciferae (Coleoptera: Chrysomelidae). Oecologia. 1972 12;10(4):321–46. 10.1007/BF00345736 28307065

[2] Letourneau DK, Armbrecht I, Rivera BS, Lerma JM, Carmona EJ, Daza MC, et al Does plant diversity benefit agroecosystems? A synthetic review. Ecol Appl Publ Ecol Soc Am. 2011 1;21(1):9–21. 10.1890/09-2026.1 21516884

[3] Barbosa P, Hines J, Kaplan I, Martinson H, Szczepaniec A, Szendrei Z. Associational Resistance and Associational Susceptibility: Having Right or Wrong Neighbors. Annu Rev Ecol Evol Syst. 2009;40(1):1–20.

[4] Hambäck PA, Inouye BD, Andersson P, Underwood N. Effects of plant neighborhoods on plant—herbivore interactions: resource dilution and associational effects. Ecology. 2014 5 1;95(5):1370–83. 25000768 10.1890/13-0793.1

[5] Agrawal AA, Lau JA, Hambäck PA. Community heterogeneity and the evolution of interactions between plants and insect herbivores. Q Rev Biol. 2006 12;81(4):349–76. 17240728 10.1086/511529

[6] Orrock JL, Holt RD, Baskett ML. Refuge-mediated apparent competition in plant—consumer interactions. Ecol Lett. 2010 1 1;13(1):11–20. 10.1111/j.1461-0248.2009.01412.x 19930397

[7] Underwood N, Inouye BD, Hambäck PA. A Conceptual Framework for Associational Effects: When Do Neighbors Matter and How Would We Know? Q Rev Biol. 2014;89(1):1–19. 24672901 10.1086/674991

[8] Stastny M, Agrawal AA. Love thy neighbor? reciprocal impacts between plant community structure and insect herbivory in co-occurring Asteraceae. Ecology. 2014 10 1;95(10):2904–14.

[9] Marquis RJ, Lill JT, Piccinni A. Effect of plant architecture on colonization and damage by leaftying caterpillars of Quercus alba. Oikos. 2002;99:531–7.

[10] Shiojiri K, Karban R. Plant age, communication, and resistance to herbivores: young sagebrush plants are better emitters and receivers. Oecologia. 2006 8;149(2):214–20. 10.1007/s00442-006-0441-0 16736187

[11] Plath M, Dorn S, Riedel J, Barrios H, Mody K. Associational resistance and associational susceptibility: specialist herbivores show contrasting responses to tree stand diversification. Oecologia. 2012 6;169(2):477–87. 10.1007/s00442-011-2215-6 22159991

[12] Morrison S, Barton L, Caputa P, Hik DS. Forage selection by collared pikas, Ochotona collaris, under varying degrees of predation risk. Can J Zool. 2004 4;82(4):533–40.

[13] Bach CE. Plant Spatial Pattern and Herbivore Population Dynamics: Plant Factors Affecting the Movement Patterns of a Tropical Cucurbit Specialist (Acalymma Innubum). Ecology. 1984;65(1):175–90.

[14] Cipollini DF, Bergelson J. Plant Density and Nutrient Availability Constrain Constitutive and Wound-induced Expression of Trypsin Inhibitors in Brassica napus. J Chem Ecol. 2001 3;27(3):593–610. 11441448 10.1023/a:1010384805014

[15] Cipollini DF, Bergelson J. Interspecific competition affects growth and herbivore damage of Brassica napus in the field. Plant Ecol. 2002 10;162(2):227–31.

[16] Agrawal AA. Resistance and Susceptibility of Milkweed: Competition, Root Herbivory, and Plant Genetic Variation. Ecology. 2004 8 1;85(8):2118–33.

[17] Kos M, Bukovinszky T, Mulder PPJ, Bezemer TM. Disentangling above- and belowground neighbor effects on the growth, chemistry, and arthropod community on a focal plant. Funct Ecol. 2015 1 1;96(1):164–75. 10.1890/14-0563.1 26236901

[18] Sato Y, Kudoh H. Associational effects against a leaf beetle mediate a minority advantage in defense and growth between hairy and glabrous plants. Evol Ecol. 2016 2 1;30(1):137–54.

[19] Karban R. Communication between sagebrush and wild tobacco in the field. Biochem Syst Ecol. 2001 11;29(10):995–1005.

[20] Kost C, Heil M. Herbivore-induced plant volatiles induce an indirect defence in neighbouring plants: Airborne volatiles induce indirect plant defence. J Ecol. 2006 2 16;94(3):619–28.

[21] Himanen SJ, Blande JD, Klemola T, Pulkkinen J, Heijari J, Holopainen JK. Birch (Betula spp.) leaves adsorb and re-release volatiles specific to neighbouring plants—a mechanism for associational herbivore resistance? New Phytol. 2010 5;186(3):722–32. 10.1111/j.1469-8137.2010.03220.x 20298484

[22] Bach CE. Effects of Microclimate and Plant Characteristics on the Distribution of a Willow Flea Beetle, Altica subplicata. Am Midl Nat. 1993;130(1):193–208.

[23] Chown SL, Nicolson SW. Insect Physiological Ecology: Mechanisms and Patterns. OUP Oxford; 2004 262 p.

[24] Feeny P. Plant Apparency and Chemical Defense In: Wallace JW, Mansell RL, editors. Biochemical Interaction Between Plants and Insects [Internet]. Springer US; 1976 [cited 2016 Apr 2]. p. 1–40. (Recent Advances in Phytochemistry). http://link.springer.com/chapter/10.1007/978-1-4684-2646-5_1

[25] Wise MJ, Abrahamson WG, Cole JA. The role of nodding stems in the goldenrod—gall—fly interaction: A test of the “ducking” hypothesis. Am J Bot. 2010 3 1;97(3):525–9. 10.3732/ajb.0900227 21622414

[26] Castagneyrol B, Giffard B, Péré C, Jactel H. Plant apparency, an overlooked driver of associational resistance to insect herbivory. J Ecol. 2013 3 1;101(2):418–29.

[27] Kim TN, Underwood N. Plant neighborhood effects on herbivory: damage is both density and frequency dependent. Ecology. 2015 5;96(5):1431–7. 26236855 10.1890/14-1097.1

[28] Verschut TA, Becher PG, Anderson P, Hambäck PA. Disentangling associational effects: both resource density and resource frequency affect search behaviour in complex environments. Funct Ecol. 2016 11;30(11):1826–1833.

[29] Hahn PG, Orrock JL. Neighbor palatability generates associational effects by altering herbivore foraging behavior. Ecology. 2016 8 1;97(8):2103–11. 10.1002/ecy.1430 27859184

[30] Rand TA. Effects of environmental context on the susceptibility of Atriplex patula to attack by herbivorous beetles. Oecologia. 1999 10;121(1):39–46. 10.1007/s004420050905 28307887

[31] Hambäck PA, Agren J, Ericson L. Associational Resistance: Insect Damage to Purple Loosestrife Reduced in Thickets of Sweet Gale. Ecology. 2000;81(7):1784–94.

[32] Letourneau DK. Associational Susceptibility: Effects of Cropping Pattern and Fertilizer on Malawian Bean Fly Levels. Ecol Appl. 1995;5(3):823–9.

[33] Orians CM, Björkman C. Associational Resistance to a Tropical Leaf-Miner: Does Neighbour Identity Matter? J Trop Ecol. 2009;25(5):551–4.

[34] Inouye BD. Response Surface Experimental Designs for Investigating Interspecific Competition. Ecology. 2001 10 1;82(10):2696–706.

[35] Kim TN, Underwood N, Inouye BD. Insect herbivores change the outcome of plant competition through both inter- and intraspecific processes. Ecology. 2013;94(8):1753–63. 24015519 10.1890/12-1261.1

[36] Kim TN. Plant damage and herbivore performance change with latitude for two old-field plant species, but rarely as predicted. Oikos. 2014 7 1;123(7):886–96.

[37] Werner PA, Gross RS, Bradbury IK. The Biology of Canadian Weeds: 45. Solidago canadensis L. Can J Plant Sci. 1980 10 1;60(4):1393–409.

[38] Wise MJ, Cummins JJ, Young CD. Compensation for floral herbivory in Solanum carolinense: identifying mechanisms of tolerance. Evol Ecol. 2007 2 24;22(1):19–37.

[39] Bosio CF, McCrea KD, Nitao JK, Abrahamson WG. Defense Chemistry of Solidago altissima: Effects on the Generalist Herbivore Trichoplusia ni (Lepidoptera: Noctuidae). Environ Entomol. 1990 6 1;19(3):465–8.

[40] Root RB. Herbivore Pressure on Goldenrods (Solidago Altissima): Its Variation and Cumulative Effects. Ecology. 1996;77(4):1074–87.

[41] Wise MJ, Sacchi CF. Impact of two specialist insect herbivores on reproduction of horse nettle, Solanum carolinense. Oecologia. 1996 10;108(2):328–37. 10.1007/BF00334658 28307846

[42] Holm S. A Simple Sequentially Rejective Multiple Test Procedure. Scand J Stat. 1979;6(2):65–70.

[43] R Development Core Team. R: a language and environment for statistical computing. Austria, Vienna 2014.

[44] Gelman A, Hill J. Data Analysis Using Regression and Multilevel/Hierarchical Models. Cambridge, MA: Cambridge University Press; 2007 625 p.

[45] Schröder R, Hilker M. The Relevance of Background Odor in Resource Location by Insects: A Behavioral Approach. BioScience. 2008 4 1;58(4):308–16.

[46] Silander JA, Pacala SW. Neighborhood Predictors of Plant Performance. Oecologia. 1985;66(2):256–63. 10.1007/BF00379863 28311598

[47] Hughes AR. A neighboring plant species creates associational refuge for consumer and host. Ecology. 2012 6;93(6):1411–20. 22834381 10.1890/11-1555.1

[48] Edwards RL, Epp HT. The Influence of Soil Moisture and Soil Type on the Oviposition Behaviour of the Migratory Grasshopper, Melanoplus sanguinipes (Fabricius). Can Entomol. 1965 4;97(04):401–409.

[49] Lapointe SL, Shapiro JP. Effect of Soil Moisture on Development of Diaprepes abbreviatus (Coleoptera: Curculionidae). Fla Entomol. 1999;82(2):291–9.

[50] Yamawo A, Hada Y, Suzuki N. Variations in direct and indirect defenses against herbivores on young plants of Mallotus japonicus in relation to soil moisture conditions. J Plant Res. 2012 1;125(1):71–6. 10.1007/s10265-011-0407-0 21331791

[51] Strong DR, Lawton JH, Southwood SR. Insects on Plants: Community Patterns and Mechanisms. Harvard University Press; 1984 313 p.

[52] McNutt D.W., Halpern S., Barrows K. and Underwood N..Intraspecific competition facilitates the evolution of tolerance to insect damage in the perennial plant Solanum carolinense. Oecologia 2012 17: 1033–1044 10.1007/s00442-012-2377-x 22684886

[53] Jactel H, Brockerhoff EG. Tree diversity reduces herbivory by forest insects. Ecol Lett. 2007 9;10(9):835–48. 10.1111/j.1461-0248.2007.01073.x 17663717

[54] Turchin P, Wood SN, Ellner SP, Kendall BE, Murdoch WW, Fischlin A, et al Dynamical Effects of Plant Quality and Parasitism on Population Cycles of Larch Budmoth. Ecology. 2003 5 1;84(5):1207–14.

[55] Hambäck PA, Englund G. Patch area, population density and the scaling of migration rates: the resource concentration hypothesis revisited. Ecol Lett. 2005 10 1;8(10):1057–65.

