# Supplementary material for: How plant neighborhood composition influences herbivory: Testing four mechanisms of associational resistance and susceptibility
Source: PLoS One. 2017 May 9;12(5):e0176499. doi: 10.1371/journal.pone.0176499 (PMC5423596; doi:10.1371/journal.pone.0176499)
Supplement: S1 Table — (PDF) [file pone.0176499.s001.pdf]

Kim 2017. How plant neighborhood composition influences herbivory: testing four mechanisms of associational resistance and susceptibility. PlosOne.

S1 Table. ANOVA tables for neighborhood composition effects on (A) *Solanum* damage, (B) herbivore load per *Solanum* plant, (C) predator load per *Solanum* plant, and (D) ratio of predator to herbivore load per *Solanum* plant.

	A. *Solanum* damage		B. Herbivore load		C. Predator load		D. Predator to herbivore load	
Variable	$F_{1,47}$	P	$F_{1,47}$	P	$F_{1,47}$	P	$F_{1,47}$	P
Total Density	1.14	0.28	1.58	0.21	8.32	<0.01	6.48	0.01
Density of *Solanum*	0.21	0.64	0.21	0.64	0.60	0.44	1.35	0.25
Frequency of *Solidago*	10.83	<0.01	0.03	0.85	2.00	0.16	1.37	0.24
