# Supplementary material for: How plant neighborhood composition influences herbivory: Testing four mechanisms of associational resistance and susceptibility
Source: PLoS One. 2017 May 9;12(5):e0176499. doi: 10.1371/journal.pone.0176499 (PMC5423596; doi:10.1371/journal.pone.0176499)
Supplement: S2 Table — (PDF) [file pone.0176499.s002.pdf]

S2 Table. ANOVA tables for neighborhood composition effects on microclimate conditions: (A) mean ambient temperature, (B) mean light intensity, and (C) mean soil moisture content.

	A. Temperature		B. Light intensity		C. Soil moisture	
Variable	$F_{1,47}$	*P*	$F_{1,47}$	*P*	$F_{1,47}$	*P*
Total Density	5.37	0.02	3.78	0.05	1.15	0.28
Density of *Solanum*	3.08	0.08	4.76	0.03	18.68	<0.01
Frequency of *Solidago*	0.10	0.75	0.38	0.53	53.55	<0.01
