# Supplementary material for: How plant neighborhood composition influences herbivory: Testing four mechanisms of associational resistance and susceptibility
Source: PLoS One. 2017 May 9;12(5):e0176499. doi: 10.1371/journal.pone.0176499 (PMC5423596; doi:10.1371/journal.pone.0176499)
Supplement: S3 Table — (PDF) [file pone.0176499.s003.pdf]

S3 Table. ANOVA tables for neighborhood composition effects on plant resistance: (A) constitutive and (B) induced resistance of *Solanum* to a specialist beetle, *Leptinotarsa juncta*.

	A. Constitutive resistance			B. Induced resistance		
Variable	*DF*	*F*	*P*	*DF*	*F*	*P*
Intercept	1	27.98	< 0.01	1	15.28	<0.01
Total density	1	1.23	0.28	1	0.63	0.44
Plant genotype	3	3.41	0.02	3	7.96	<0.01
Solidago frequency	1	4.56	0.03	1	4.33	0.05
Solanum density	1	2.64	0.19	1	1.87	0.19
Total density: Plant genotype	3	3.59	0.06	3	3.18	0.06
Solidago frequency: Plant genotype	3	4.49	0.04	3	4.38	0.02
Solanum density: Plant genotype	3	3.22	0.01	3	3.56	0.04
