# Supplementary material for: How plant neighborhood composition influences herbivory: Testing four mechanisms of associational resistance and susceptibility
Source: PLoS One. 2017 May 9;12(5):e0176499. doi: 10.1371/journal.pone.0176499 (PMC5423596; doi:10.1371/journal.pone.0176499)
Supplement: S1 Text — (PDF) [file pone.0176499.s004.pdf]

Kim 2017. How plant neighborhood composition influences herbivory: testing four mechanisms of associational resistance and susceptibility. PlosOne.

S1 Text. Neighborhoods used for pair-wise preference experiments.

Neighborhoods used for pair-wise preference experiments. Neighborhoods denoted as "*X,Y* ". *X* is the number of *Solanum*, *Y* is the number of *Solidago* (see Fig. 2 for response surface). Four types of comparisons were made to examine which component of the neighborhood influenced preference. (A) First, to examine how plant density influenced neighborhood preference, I used neighborhoods where total plant densities were held constant at 4 or 16 individual plants ("low" and "high" total densities, respectively) but the densities of *Solanum* and *Solidago* varied (a substitutive design comparison at two total density levels). For each paired treatment, preference for a particular neighborhood type was recorded. A substitutive design comparison allowed me to determine whether variation in plant density influenced preference but cannot tell me whether preference is due to the density of *Solanum* or *Solidago* because both are changing simultaneously. To overcome this, I made another set of pairwise neighborhood comparisons (B) where densities of *Solanum* remained fixed at either one, four, and eight individuals and compared to neighborhoods with the same amount of *Solanum* but the density of *Solidago* varied at 15, 12, and 8 individuals respectively. Because this additive design comparison kept the density of *Solanum* constant but varied the density of *Solidago*, I was able to determine whether preference was due to *Solidago* density. Again, for each paired treatment, preference for a particular neighborhood type was recorded. Finally, because paired neighborhoods in the previous additive comparisons also varied in size (thus confounding total density and the density *Solidago*), a third set of pair-wise comparisons was made between monocultures of varying sizes (one, four, 16 individuals) to determine if neighborhood size alone influenced preference. For each paired treatment, preference for a particular neighborhood type was recorded.

A. Substitutive comparisons (fixed *total* density)

Low density (4 individuals)

4,0	vs.	2,2
2,2	vs.	1,3
1,3	vs.	4,0

High density (16 individuals)

16,0	vs.	8,8
8,8	vs.	1,15
1,15	vs.	16,0

B. Additive comparisons (fixed *Solanum* densities)

Monoculture		*Diculture*
1,0	vs.	1,15
4,0	vs.	4,12
8,0	vs.	8,8

C. Neighborhood size (monoculture of *Solanum* only)

1,0	vs.	4,0
4,0	vs.	16,0
16,0	vs.	1,0
